# Annotation-efficient, patch-based, explainable deep learning using curriculum method for breast cancer detection in screening mammography

**DOI:** 10.1186/s13244-025-01922-w

**Published:** 2025-03-19

**Authors:** Ozden Camurdan, Toygar Tanyel, Esma Aktufan Cerekci, Deniz Alis, Emine Meltem, Nurper Denizoglu, Mustafa Ege Seker, Ilkay Oksuz, Ercan Karaarslan

**Affiliations:** 1 Department of Radiology, Acibadem Healthcare Group, Istanbul, Turkey; 2 Biomedical Engineering Graduate Program, Istanbul Technical University, Istanbul, Turkey; 3 Department of Radiology, Sisli Hamidiye Etfal Training and Research Hospital, Istanbul, Turkey; 4 Department of Radiology, School of Medicine, Acibadem Mehmet Ali Aydinlar University, Istanbul, Turkey; 5 Department of Radiology, University of Health Sciences Istanbul Training and Research Hospital, Istanbul, Turkey; 6 Department of Radiology, Sultan 2. Abdulhamid Han Training and Research Hospital, Istanbul, Turkey; 7 School of Medicine, Acibadem Mehmet Ali Aydinlar University, Istanbul, Turkey; 8 Department of Computer Engineering, Istanbul Technical University, Istanbul, Turkey

**Keywords:** Breast cancer detection, Curriculum learning, Deep learning, Explainable artificial intelligence (XAI), Mammography

## Abstract

**Objectives:**

To develop an efficient deep learning (DL) model for breast cancer detection in mammograms, utilizing both weak (image-level) and strong (bounding boxes) annotations and providing explainable artificial intelligence (XAI) with gradient-weighted class activation mapping (Grad-CAM), assessed by the ground truth overlap ratio.

**Methods:**

Three radiologists annotated a balanced dataset of 1976 mammograms (cancer-positive and -negative) from three centers. We developed a patch-based DL model using curriculum learning, progressively increasing patch sizes during training. The model was trained under varying levels of strong supervision (0%, 20%, 40%, and 100% of the dataset), resulting in baseline, curriculum 20, curriculum 40, and curriculum 100 models. Training for each model was repeated ten times, with results presented as mean ± standard deviation. Model performance was also tested on an external dataset of 4276 mammograms to assess generalizability.

**Results:**

F1 scores for the baseline, curriculum 20, curriculum 40, and curriculum 100 models were 80.55 ± 0.88, 82.41 ± 0.47, 83.03 ± 0.31, and 83.95 ± 0.55, respectively, with ground truth overlap ratios of 60.26 ± 1.91, 62.13 ± 1.2, 62.26 ± 1.52, and 64.18 ± 1.37. In the external dataset, F1 scores were 74.65 ± 1.35, 77.77 ± 0.73, 78.23 ± 1.78, and 78.73 ± 1.25, respectively, maintaining a similar performance trend.

**Conclusion:**

Training DL models with a curriculum method and a patch-based approach yields satisfactory performance and XAI, even with a limited set of densely annotated data, offering a promising avenue for deploying DL in large-scale mammography datasets.

**Critical relevance:**

This study introduces a DL model for mammography-based breast cancer detection, utilizing curriculum learning with limited, strongly labeled data. It showcases performance gains and better explainability, addressing challenges of extensive dataset needs and DL’s “black-box” nature.

**Key Points:**

- Increasing numbers of mammograms for radiologists to interpret pose a logistical challenge.
- We trained a DL model leveraging curriculum learning with mixed annotations for mammography.
- The DL model outperformed the baseline model with image-level annotations using only 20% of the strong labels.
- The study addresses the challenge of requiring extensive datasets and strong supervision for DL efficacy.
- The model demonstrated improved explainability through Grad-CAM, verified by a higher ground truth overlap ratio.
- He proposed approach also yielded robust performance on external testing data.

**Graphical Abstract:**

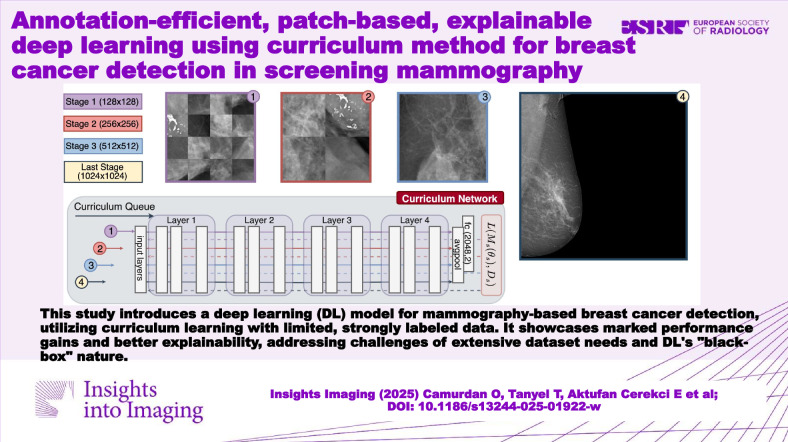

## Introduction

Mammographic screenings are pivotal in the early detection of breast cancer, attributed to their accessibility, cost-efficiency, and dependable accuracy in spotting abnormalities [1, 2]. Nevertheless, the growing need for analyzing mammograms presents considerable challenges for radiologists. It includes delays in report generation, missed screenings, and a heightened risk of diagnostic inaccuracies [3–6].

In the last few years, the evolution of hardware and software and the rapid increase in digital medical data have elevated deep learning (DL) as a viable approach. DL aids healthcare professionals in diagnosing various diseases and managing numerous tasks [7–10]. This technology has significantly impacted mammography, with several studies demonstrating that DL can perform on par with or surpass radiologists in detecting breast cancer [11, 12].

DL models in mammography require extensive training on large datasets to achieve accuracy comparable to that of radiologists. [13–15]. Another characteristic of DL models, especially in computer vision, is their improved performance under supervised training regimes, where ground-truth labels are provided. This enhancement is even more pronounced with solid supervision involving pixel-level or bounding-box annotations, as opposed to weak supervision that relies on image-level or scan-level annotations.

A notable challenge with training DL models using weak-level annotations is the generation of “black-box” or “opaque” outputs [12]. Efforts to mitigate this issue have led to the development of various techniques under the umbrella of explainable artificial intelligence (XAI) [16].

While it is relatively straightforward to acquire scan-level annotations for medical scans, for instance, mammography is more complex by employing natural language processing on radiology reports. Given the high resolution of mammography scans and the typically small size of lesions, strong annotations (such as precise lesion markings) could significantly enhance model performance. However, this approach requires the involvement of expert radiologists, whose time is already a scarce resource [17].

This scarcity is, in fact, a primary motivator for applying DL technologies to mammography screening for breast cancer diagnosis in the first place [18]. Therefore, developing data-efficient training methods that combine strong- and weak-level supervision could be highly beneficial.

In this work, we designed a DL model trained with patches and the curriculum learning method. We compared its performance with a baseline DL model trained on image-level annotations for detecting breast cancer. We tested the proposed approach on an external dataset (*n* = 4276) of mammography examinations from a different center. Additionally, we explored whether the proposed model yields better explainability using gradient-weighted class activation mapping (Grad-CAM) assessed quantitatively by the ground truth overlap ratio [19–21].

## Methods

### Study sample

This study was conducted with approval from the local ethics committee, which also waived the need for informed consent due to the retrospective analysis of de-identified medical records. We utilized data from three affiliated private university hospitals, examining records from the hospital information system and picture archive and communication system from January 2020 to January 2022. The study focused on adult women who underwent opportunistic screening mammography, totaling 18233 women.

All examinations were 2D digital mammography examinations, including craniocaudal (CC) and mediolateral oblique (MLO) views of each breast. The mammograms were acquired using Senographe Essential (GE Healthcare, Chicago, IL, USA), Lorad Selenia, and Selenia Dimensions (Hologic Inc., Marlborough, MA, USA). We excluded patients with a history of breast surgery, radiotherapy, or chemotherapy prior to their screening mammogram, resulting in 106 exclusions.

We then selected women diagnosed with breast cancer through subsequent pathology following their screening, forming our breast cancer sample (*n* = 988). Our selection included all lesion types identified in breast cancer, including masses, microcalcifications, or combinations thereof, to mirror the diversity of clinical presentations.

For the control group, we included women who received a breast imaging-reporting and data system (BI-RADS) score of 1 or 2, indicating no suspicion of cancer, and who had either undergone a follow-up screening at least a year later or had benign pathology confirmed. To align the numbers between the groups for our analyses, we reduced the size of the control group from 17,245 to 988 to equal that of the breast cancer group, recognizing the potential for bias this may introduce.

We addressed the bias from under-sampling through stratified random sampling, ensuring the control group accurately reflected the broader population’s characteristics, including age and BI-RADS scores. The study’s dataset was then divided into a development set, which included training and validation subsets, and a held-out internal testing set, using a 75%/25% division.

For the external validation, we gathered mammography examinations of women obtained between January 2018 and January 2020 at a state hospital. We selected women (*n* = 4276) who were diagnosed with breast cancer through subsequent pathology following their screening, forming our breast cancer sample (*n* = 2138). The mammograms were acquired using MAMMOMAT Revelation (Siemens Healthineers, Erlangen, Germany). By the development and internal testing, the number of breast cancer-negative examinations in the external sample was also 2138. In the external validation sample, the breast cancer-negative examinations had a BI-RADS score of 1 or 2, indicating no suspicion of cancer. These individuals had undergone a follow-up screening at least two years later or had benign pathology confirmed.

The study adhered to the “Studies of Diagnostic Accuracy (STARD)” reporting guideline [22], and the fifth Edition of BI-RADS of the American College of Radiology (BI-RADS) was followed [23].

### Ground truthing

Ground truth annotations were carried out by a team of three board-certified breast radiologists, each with over five years of experience in breast imaging. The radiologists annotated mammograms from the breast cancer group using a specialized workstation that included a browser-based annotation tool (https://matrix.md.ai) and a 6-megapixel diagnostic monitor (Radiforce RX 660, EIZO). All mammograms were reviewed in the digital imaging and communications in medicine format.

The process began with a thorough examination of the mammograms from the group without breast cancer, cross-checking these images against corresponding clinical and pathology reports to verify their status as true negatives. The radiologists worked in real-time through a virtual meeting platform, simultaneously reviewing each mammogram while discussing the presence and location of breast cancer. The annotations were made by consensus, with discussions centered on where and whether to place bounding boxes around potential cancerous areas. If there was a disagreement, a majority decision (2 out of 3 radiologists) was adopted as the final reference.

While adhering to the BI-RADS guidelines, radiologists labeled image regions containing lesions, allowing for a minimal inclusion of healthy tissue to account for a slight error margin. When a mammogram showed more than one cancerous lesion, each was marked with its bounding box. These annotations were applied to both the CC and MLO views to document the location and size of the cancerous lesions fully.

An independent ground truth verification was not pursued, as the study did not aim to evaluate inter-rater reliability.

### Data preprocessing and patch creation

To prepare the mammograms for analysis, we developed a specialized algorithm to remove unnecessary space around the breast tissue, focusing the images on the relevant areas. The method involves running a patch-based analysis across the entire mammogram, systematically scanning for relevant regions of breast tissue. Each patch—a square piece of the image—is evaluated, allowing the algorithm to distinguish areas containing breast tissue from irrelevant regions such as empty zero-pixel areas or the belly. Once the relevant areas are identified, a bounding box is drawn around the most significant section of breast tissue to ensure no critical diagnostic information is excluded.

The optimal parameters for this process were a patch size of 400 pixels, a stride of 80, and a black threshold of 30%. After cropping and padding the images to make them square, all images are resized to consistent 1024 × 1024 pixels, with corresponding adjustments to the bounding boxes to reflect the scaled dimensions accurately. This resolution was selected based on the standard post-cropping dimensions of the mammograms, which vary by view. By padding the images to ensure they are square before resizing, we preserved the aspect ratio and avoided distortion, ensuring that critical diagnostic features were retained.

After resizing, a radiologist carefully checked each image to confirm no crucial information was lost during the cropping and resizing process. This quality control is vital to ensure the images were prepared correctly for accurate analysis.

Next, we further divided these prepared images into smaller patches. These are square pieces of the image, created in three different sizes: 128 × 128, 256 × 256, and 512 × 512 pixels. This approach, starting from the largest size of 512 × 512 and progressively reducing the patch size, also allowed us to cover smaller regions of interest. Patch sizes below 128 × 128 were excluded due to the impractical number of patches generated—reaching millions to billions—making reliable training infeasible. These three patch sizes helped the DL model focus on specific areas of the image while maintaining a balance between covering sufficient image detail and computational feasibility.

**Fig. 1:**
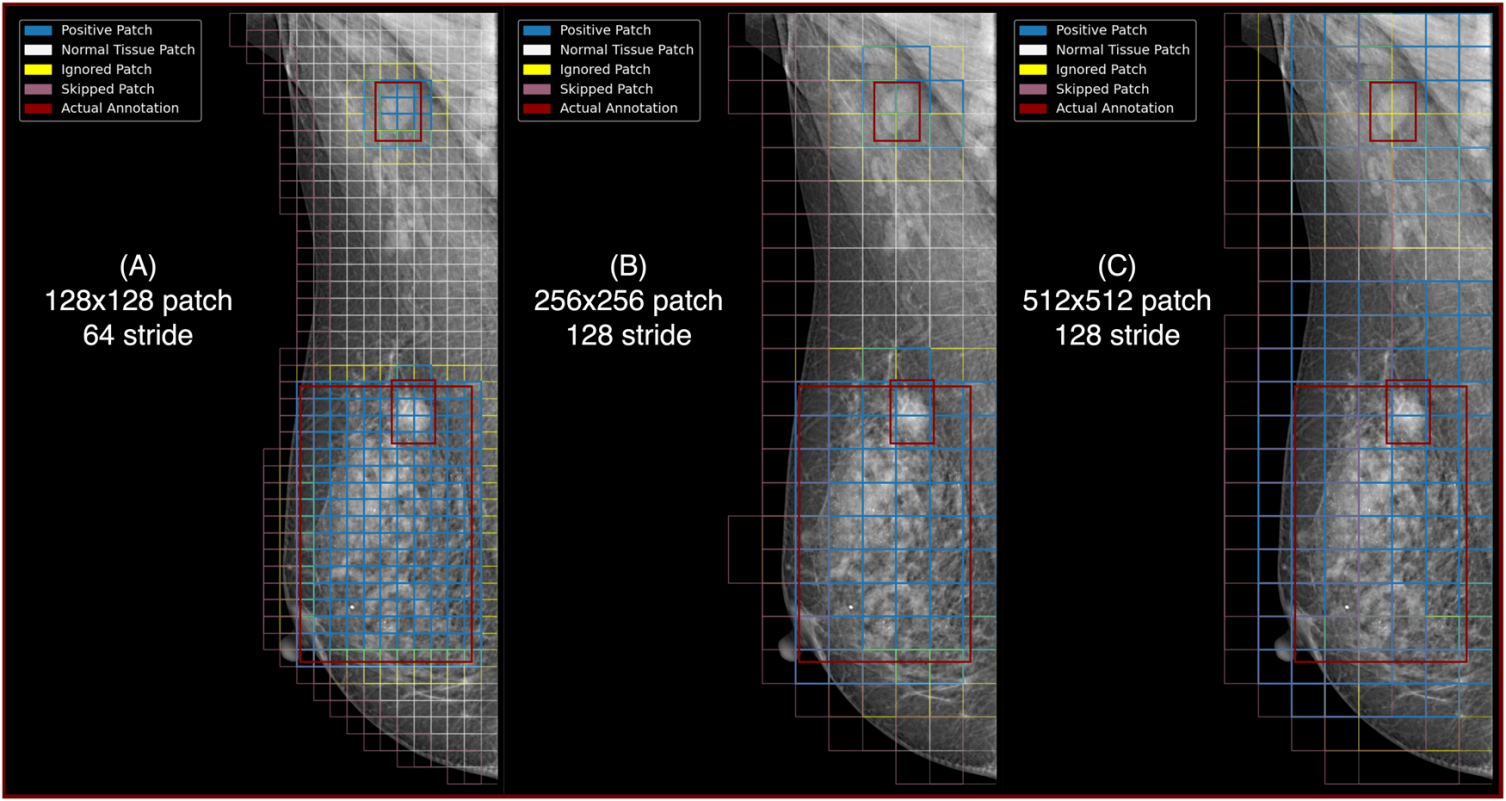
Illustration of the different types of patches used in the DL pipeline. Positive patches, which overlap with bounding boxes and contain suspicious tissue indicative of potential cancer; negative patches, which display normal tissue and show no signs of cancer; ignored patches, which have insufficient overlap with bounding boxes to be definitively classified as either positive or negative; and skipped patches, which are located at the periphery of the breast and are omitted for computational efficiency. The term “actual annotation” refers to the ground-truth reference against which these patches are evaluated

We used a set of known cancerous areas, marked by bounding boxes, to identify which patches likely contained signs of cancer. A patch was considered to show signs of cancer (positive) if more than half of it overlapped with these marked cancerous areas. Conversely, patches showing little overlap (less than 10%) were deemed unlikely to contain cancer (negative). Patches with 10% and 50% overlaps were not used for training to keep the data precise and prevent confusion in the learning process. Figure 1 shows how these patches were selected and categorized.

### DL model

Our methodology employed a multi-scale, patch-based pretraining strategy using the ResNeXt50_32x4d model, chosen for its scale-invariant characteristics. This model supports training across diverse image sizes, thanks to its adaptive CNN layers and an AdaptiveAvgPool layer for final pooling. Experiments were executed using PyTorch on an NVIDIA L4 GPU.

### Curriculum learning pipeline

Our training method, curriculum learning, is divided into four progressive stages. Initially, the DL model is trained on small sections of the mammogram, called patches, which are 128 × 128 pixels. As training progresses, the size of these patches increases—first to 256 × 256 pixels and then to 512 × 512 pixels. This step-by-step approach helps the model focus first on small, detailed features of the image, which are crucial for identifying signs of breast cancer.

In the final stage of training, we introduce the model to the entire mammogram image. This stage uses a more extensive view to help the model understand how the detailed features it learned earlier fit into the bigger picture. Moving from smaller to more extensive views, the model learns to recognize cancer signs up close and at a more general level.

**Fig. 2:**
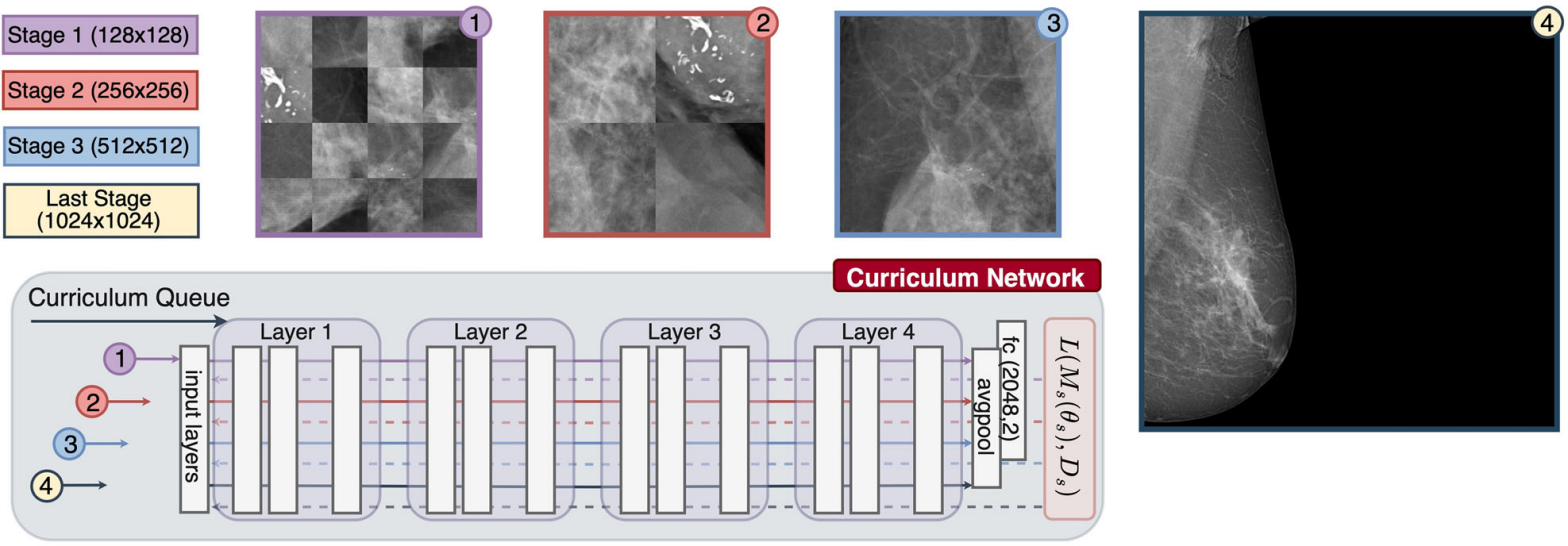
Proposed DL framework. This framework adopts the ResNeXt50_32x4d architecture, characterized by its user-configurable stages and properties, adjustable through a configuration file. In the baseline configuration, the network exclusively processes full-sized images at its final stage. In contrast, the curriculum learning approach modifies this process; the network initially handles the first three stages using patches of images, culminating in processing full-sized images in the final stage, all within the same network framework

This gradual training process aims to teach the model systematically, starting with detailed observations and moving toward a more comprehensive understanding. This method improves the model’s accuracy and ensures it can interpret various scales of image details, from very small to the entire image. Figure 2 in our paper visually explains this curriculum learning process.

### Annotation-efficient approach

Our study used different training strategies to make our model efficient in learning from annotations. Annotations are crucial marks on images that tell the model exactly where to look for signs of breast cancer.

#### Curriculum 100 model

This version of our model used all available annotated data. We applied our curriculum learning method, which starts with small image patches and gradually introduces larger ones. This model serves as a reference point for evaluating other models, using the most comprehensive data set for training.

#### Curriculum 20 and curriculum 40 models

These models were trained on a reduced amount of detailed annotations—20% and 40% of the data, respectively. The idea is to see how well the model can perform when it has fewer specific guidelines (patches). For the rest of the training, these models used full images with essential labels, which provide less detail but cover more general features.

#### Baseline model

The baseline model was trained using only these less detailed, image-level labels. This approach uses the most basic form of supervision and tests the model’s learning ability with little specific guidance.

Each of these models produces predictions for whether a mammogram shows signs of breast cancer. These predictions are then averaged with other relevant data from the same side of the body (ipsilateral breast) to determine the presence of cancer. This strategy allows us to compare how different levels of detail in training data affect the model’s ability to diagnose breast cancer accurately.

### Model training

We trained our model over several phases to ensure it learns effectively from mammogram images. Initially, the model was trained in three stages, each consisting of 100 cycles known as epochs, at a fixed learning rate of 1e-05. These stages used increasingly more significant sections of the images, allowing the model to progressively improve its ability to detect features indicative of breast cancer. After completing these phases, we extended the training for 15 epochs using full-sized images to help the model apply its learned skills to complete images.

We employed the cross-entropy loss function to measure the model’s performance, which is effective in training classification models. We used the Adam optimizer for optimization, which is known for its efficiency in enhancing the learning process. We applied standard data augmentation techniques such as random flips and rotations to the training data to ensure the model could handle image orientation and position variations. However, the validation and testing images were left unchanged to ensure accuracy during final evaluations.

The entire training process was repeated ten times using the same settings to confirm the reliability of our results, with outcomes reported as averages along with standard deviations to assess consistency and stability. This thorough training approach equips the model to accurately detect breast cancer by learning from various scenarios and image presentations.

### XAI

In this study, we utilized Grad-CAM [19], a widely used explanation method for convolutional neural networks (CNNs). Grad-CAM generates ‘visual explanations’ for decisions from a broad range of CNN-based models by using the gradient information flowing into the final convolutional layer of the CNN. This approach helps to understand the contribution of each neuron to a decision of interest. The resulting heatmaps, known as Grad-CAMs, highlight the regions in the input image that were influential for the network’s output, providing insight into the model’s decision-making process.

In our study, we employed the ground truth overlap ratio [19, 20] as a quantitative measure to evaluate the performance of XAI in aligning with clinical ground truths. The primary focus was on assessing the correlation between the saliency maps produced by these methods and the ground-truth bounding boxes delineated by radiologists on mammogram lesions suspected of harboring breast cancer.

#### Statistical analyses

Statistical analyses were conducted using Python Version 3’s SciPy library. The DL model’s performance in identifying breast cancer is measured at a breast level using recall, precision, and F1 scores. For the F1 score, recall, and precision, we reported the mean and standard deviations of the ten training runs for each model. For the explainability, we assessed the performance of saliency maps using the ground truth overlap ratio as follows:

$${{Ground}\; {truth}\; {overlap}\; {ratio}}\,=\,\frac{{{\mbox{Sum}}}\, {{\mbox{of}}} \, {{\mbox{overlapping}}} \, {{\mbox{pixels}}}}{{{\mbox{Total}}}\, {{\mbox{ground}}} \, {{\mbox{truth}}} \, {{\mbox{pixels}}}}$$

The ground truth overlap ratio of 100 indicates excellent explainability, while 0 indicates worst explainability. Readers should bear in mind that the ground truth overlap ratio was calculated only for patients with cancer as the model in this study was trained to detect cancer lesions while treating patients with benign lesions or negative mammograms (BI-RADS 1-2) as negative cases (i.e., no bounding boxes are provided).

## Results

A total of 1976 women with a median age of 52 (IQR, 9) constituted the final study sample. Among these women, 988 (50%) had pathology-proven breast cancer, while the remaining 988 (50%) presented with negative mammograms—only a single mammography scan was included per patient in the present work. The development sample included 1482 women (75%), while the remaining 494 women (25%) formed the held-out internal testing set used for the unbiased evaluation of the DL methods.

As we hypothesized, the Curriculum 100 Model demonstrated superior performance, achieving the highest F1 score of 83.95 ± 0.5. In contrast, the baseline model, which received only weak supervision during training, recorded the lowest F1 score of 80.55 ± 0.88. Notably, the Curriculum 20 and 40 Models, designed to be annotation-efficient, surpassed the baseline with F1 scores of 82.41 ± 0.47 and 83.03 ± 0.31, respectively. These increments reflect the value of progressively structured training regimens that introduce complexity in a controlled manner.

**Fig. 3:**
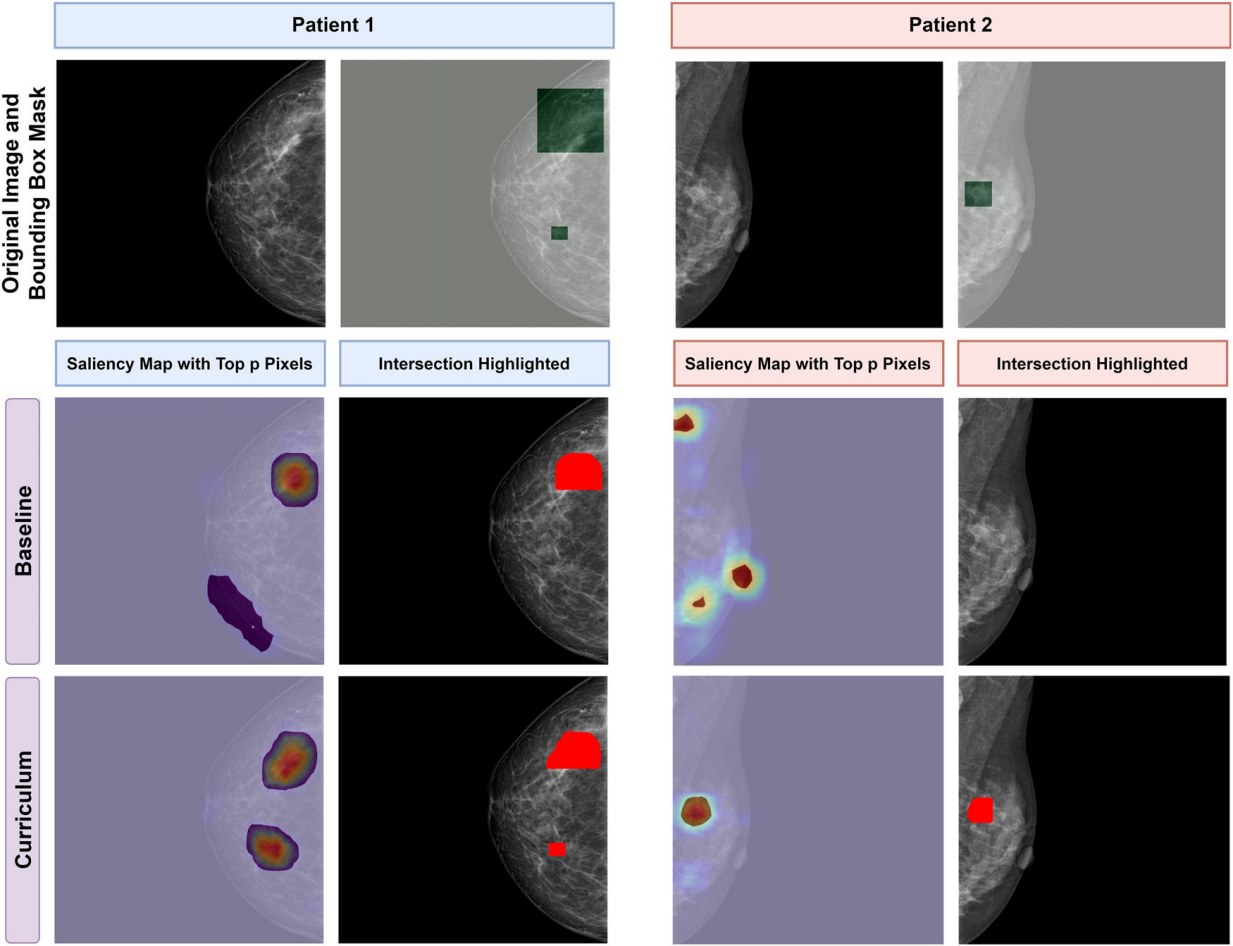
Two women with breast cancer. In comparing model success in detecting breast cancer, the baseline model incorrectly classified the image of the patients as negative, while the Curriculum 20 model correctly identified the cancer. The explainable AI approach revealed that the baseline model focused on irrelevant areas, including the nipple, whereas the Curriculum model concentrated on the cancerous lesion. The ground truth overlap ratio, calculated using the intersection of ground truth boxes and the saliency maps with top pixels, was used to evaluate the models’ performance

The Curriculum 100 Model also attained the highest ground truth overlap ratio at 64.18 ± 1.37, indicating that the predictions of this model have a higher concordance with expert annotations than the baseline model, which scored 60.26 ± 1.91 in this metric. Curriculum 20 and 40 Models surpassed the baseline with a ground truth overlap ratio of 62.13 ± 1.2 and 62.26 ± 1.52, respectively. The overlap ratio is critical as it underscores the explainability of the model’s decisions, a crucial aspect when considering integrating such systems into clinical practice. Figure 3 displays representative predictions of the baseline and Curriculum 20 models on the internal testing set. Table 1 details the performance and XAI metrics of the models.

**Table 1:** Performance and explainability metrics of DL models for breast cancer detection in mammography on the testing set

Models		Metrics		
	Ground truth overlap ratio	Recall	Precision	F1 score
Baseline	60.26 ± 1.91	80.55 ± 0.87	80.58 ± 0.86	80.55 ± 0.88
Curriculum 20	62.13 ± 1.2	82.44 ± 0.44	82.98 ± 0.7	82.41 ± 0.47
Curriculum 40	62.26 ± 1.52	83.08 ± 0.69	83.47 ± 0.14	83.03 ± 0.31
Curriculum 100	**64.18** **±** **1.37**	**84.01** **±** **0.53**	**84.49** **±** **0.39**	**83.95** **±** **0.55**

Bold indicates the best results across rows

In the external dataset, the Curriculum 100 model outperformed the others, achieving the highest F1 score of 78.73 ± 1.25, aligning with the results from the primary test set. The baseline model recorded the lowest F1 score at 74.65 ± 1.35. Notably, the Curriculum 20 and 40 Models also surpassed the baseline, with F1 scores of 77.77 ± 0.73 and 78.23 ± 1.78, respectively. Further details of the performance and XAI metrics in the external testing set are provided in Table 2.

**Table 2:** Performance metrics of DL models for breast cancer detection in mammography on the external testing set

Models		Metrics		
	Ground truth overlap ratio	Recall	Precision	F1 score
Baseline	57.2 ± 1.7	75.29 ± 1.26	78.07 ± 1.29	74.65 ± 1.35
Curriculum 20	60.53 ± 0.94	77.95 ± 0.74	79.3 ± 1.04	77.7 ± 0.73
Curriculum 40	61.02 ± 1.3	78.55 ± 2.57	**80.25** ± **0.55**	78.23 ± 1.78
Curriculum 100	**62.88** **±** **1.57**	**78.87** **±** **1.33**	79.71 ± 1.85	**78.73** **±** **1.25**

Bold indicates the best results across rows

## Discussion

The effectiveness of DL applications in breast cancer diagnosis largely hinges on intense supervision and access to extensive datasets. This demand necessitates that breast imaging specialists dedicate their scarce time to detailed annotations, such as segmentation or bounding boxes, on mammography scans. Although acquiring scan-level annotations might be more feasible via electronic health records, models trained with weak supervision often need to improve performance relative to their intensely supervised counterparts. Moreover, such models may lack transparency in their decision-making processes, especially when diagnosing a scan as indicative of breast cancer.

In response to these challenges, our study explores a patch-based DL model trained via a curriculum learning approach. This method allows us to leverage both broadly available breast-level annotations and precise bounding boxes provided by experts. Remarkably, our DL model, utilizing merely 20% of the dense annotations, outperformed the baseline model trained solely on image-level annotations. As anticipated, the model’s performance improved by integrating more densely annotated data, thus facilitating a balance between model accuracy and the time investment experts require for data annotation.

Moreover, we observed enhanced explainability in our patch-based DL model by applying the Grad-CAM method. Higher ground truth overlap ratio scores objectively validated it. Similar to the model’s performance, these scores increased with the employment of more densely annotated data, once again allowing for negotiation between performance gains and the time demands placed on expert annotators.

To assess generalizability, we further tested the model’s performance on an external dataset consisting of mammography examinations obtained at a state hospital. It contrasted with the examinations from the development set, which were obtained at university-affiliated centers. Our approach consistently demonstrated similar behavior across these different settings, further enhancing the robustness of the proposed DL pipeline.

DL methodologies have been extensively applied to breast cancer detection in mammography, reflecting diverse approaches within the literature. These methodologies span single image analyses, multi-view strategies, and multi-stage frameworks for detection and classification, alongside the adoption of various DL architectures and commercial solutions. The application of in-house and public datasets in these studies has been rigorously reviewed, as demonstrated by numerous systematic reviews [24–26]. Regardless of the varied methodologies employed, the robustness of DL models in the context of breast cancer detection on mammography, along with their potential to serve either as standalone tools or as adjuncts to radiologists, has been consistently affirmed by recent meta-analyses [25, 26].

Given this backdrop, our study does not aim to surpass existing benchmarks in breast cancer detection using DL nor to evaluate the incremental value DL models provide to radiologists in this domain—topics that have been well-established and now call for multi-center, large-scale prospective studies as underscored by recent meta-analyses. Instead, our research pivots towards investigating the annotation requirements for DL in mammography, a relatively underexplored yet critical aspect of enhancing DL model training and application.

In one of the few studies, Lotter et al introduced an annotation-efficient DL model tailored for breast cancer detection in both mammography and digital breast tomosynthesis [27]. Their approach, aligning with the methodologies employed in the current study, utilized a patch-based strategy. They incrementally trained their DL models, transitioning from intense supervision (i.e., dense annotations) to weak supervision (i.e., image-level annotations) across three distinct stages. By leveraging a mix of public and private datasets for training and evaluation, they also benchmarked their model’s performance against that of radiologists. The authors demonstrated superior generalizability of their model to populations with low screening rates, outperforming five breast-imaging specialists with an average sensitivity increase of 14%.

However, while Lotter et al confirmed the feasibility and robustness of utilizing both dense and sparse labels for training their DL model, they needed to systematically explore the model’s performance across varying ratios of vital to weak supervision [27]. In contrast, our study methodically designs an experimental framework to evaluate DL model performance at different ratios of intensely supervised training utilizing a curriculum learning approach. It allows for a direct comparison between models trained under entirely weak supervision and those under full dense supervision.

Moreover, although Lotter et al indicated that their weakly supervised model preserved localization-based interpretability in its concluding phase, they did not quantitatively validate this assertion [27]. Our research addresses this gap by demonstrating the enhanced explainability of our DL model compared to the baseline model, as evidenced by an increase in the ground truth overlap ratio that correlates with a higher ratio of dense labels.

In interpreting our study’s results, several limitations should be considered. First, given the retrospective design of our study, we recognize the inherent limitations, such as control over variables and potential biases. Second, the sample size employed in our study was relatively small, derived from three university hospital centers, potentially affecting the generalizability of our findings. To address these limitations, it is crucial to conduct further prospective studies that can validate our findings with a larger sample size in a real-world clinical setting.

Second, not all women in the control group had pathology results, as this group included those who received a BI-RADS score of 1 or 2 and underwent at least one follow-up screening a year later. However, current guidelines consider a year-long follow-up interval adequate for detecting any anomalies that may develop after the initial screening [1, 25].

Third, this study was confined to specific saliency-based methods and covered only some available techniques within the domain. This choice may have led to an incomplete picture of the performance landscape for XAI in medical imaging. While Grad-CAM was used to enhance the explainability of our model, it also has limitations [28]. Grad-CAM’s heatmaps highlight broad regions of the image, which may need more granularity for precise identification of small, subtle features. This reduced specificity can impact the model’s interpretability, particularly for tasks requiring fine detail, such as detecting early signs of cancer. Similarly, our analysis was limited to CNNs and did not extend to vision transformers or attention-based methods. This restriction could limit the applicability of our findings to the broader range of emerging methods in DL for medical imaging.

Fourth, while our current findings are promising, they primarily focus on the overall performance without differentiating between mammographic views such as CC and MLO. Future studies could explore the impact of curriculum learning on these specific views to assess whether the approach benefits one view more significantly than the other. Such analysis would validate our method further and enhance its clinical applicability by tailoring model training to the characteristics of each view.

In conclusion, the proposed annotation-efficient patch-based DL model, trained with a curriculum approach, can deliver robust performance even when using as few as 20% of the densely annotated samples from the entire training set, compared to the baseline approach which relies solely on image-level weak annotations. Furthermore, the model’s superior performance was also validated on an external dataset compared to the baseline model. Additionally, the proposed model provided improved explainability, as evidenced by the quantitative ground truth overlap ratio. Our model holds promise for enabling large-scale multi-center scans, even when only limited dense annotations are available.

## Data Availability

The datasets generated during and/or analyzed during the current study are available from the corresponding author upon reasonable request.

## Abbreviations

BI-RADS: Breast imaging-reporting and data system
CC: Craniocaudal
CNN: Convolutional neural network
DL: Deep learning
Grad-CAM: Gradient-weighted class activation mapping
MLO: Mediolateral oblique
XAI: Explainable artificial intelligence
